# The PGPR *Bacillus aryabhattai* promotes soybean growth *via* nutrient and chlorophyll maintenance and the production of butanoic acid

**DOI:** 10.3389/fpls.2024.1341993

**Published:** 2024-02-19

**Authors:** Bong-Gyu Mun, Adil Hussain, Yeon-Gyeong Park, Sang-Mo Kang, In-Jung Lee, Byung-Wook Yun

**Affiliations:** ^1^Department of Applied Biosciences, College of Agriculture and Life Sciences, Kyungpook National University, Daegu, Republic of Korea; ^2^Department of Environmental and Biological Chemistry, Chungbuk National University, Cheongju, Republic of Korea; ^3^Department of Agriculture, Abdul Wali Khan University, Mardan, Khyber Pakhtunkhwa, Pakistan

**Keywords:** plant growth promoting rhizobacteria, *Bacillus aryabhattai*, chlorophyll, butanoic acid, SRB02

## Abstract

Plant growth-promoting rhizobacteria (PGPR) colonize plant roots, establish a mutualistic relationship with the plants and help them grow better. This study reports novel findings on the plant growth-promoting effects of the PGPR *Bacillus aryabhattai*. Soil was collected from a soybean field, PGPR were isolated, identified, and characterized for their ability to promote plant growth and development. The bacterium was isolated from the soybean rhizosphere and identified as *B. aryabhattai* strain SRB02 *via* 16s rRNA sequencing. As shown by SEM, the bacterium successfully colonized rice and soybean roots within 2 days and significantly promoted the growth of the GA-deficient rice cultivar Waito-C within 10 days, as well as the growth of soybean plants with at least six times longer shoots, roots, higher chlorophyll content, fresh, and dry weight after 10 days of inoculation. ICP analysis showed up to a 100% increase in the quantity of 18 different amino acids in the SRB02-treated soybean plants. Furthermore, the 2-DE gel assay indicated the presence of several differentially expressed proteins in soybean leaves after 24 hrs of SRB02 application. MALDI-TOF-MS identified β-conglycinin and glycinin along with several other proteins that were traced back to their respective genes. Analysis of bacterial culture filtrates *via* GCMS recorded significantly higher quantities of butanoic acid which was approximately 42% of all the metabolites found in the filtrates. The application of 100 ppm butanoic acid had significantly positive effects on plant growth *via* chlorophyll maintenance. These results establish the suitability of *B. aryabhattai* as a promising PGPR for field application in various crops.

## Graphical Abstract

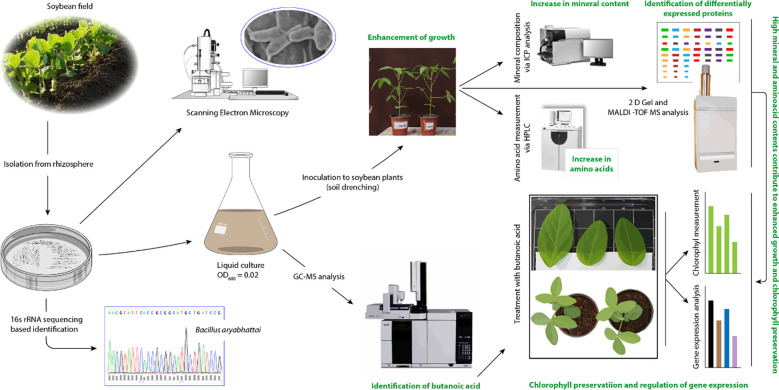

## Highlights

- The plant growth promoting rhizobacterium *Bacillus aryabhattai* strain SRB02 promotes the growth of rice and soybean plants
- SRB02 improves plant growth *via* nutrient and chlorophyll maintenance and the production of butanoic acid.
- SRB02 improves plant growth by regulating the expression of key growth-related genes.

## Introduction

Plants in their natural ecosystem, interact with other organisms that make up the bulk of soil flora and fauna. However, not all of these interactions are advantageous for the plants. Most of the interactions occur between plant roots and soil-born phytopathogens. Yet, other interactions prove to be quite beneficial for the plants. Some bacteria that live in the root zone (rhizosphere) of plants can colonize and interact with plant roots. These bacteria are called rhizobacteria, but this term usually refers to the ones that have a mutualistic or symbiotic relationship with the plants and help them grow better. These bacteria are also known as plant growth-promoting rhizobacteria or PGPR (Vessey, 2003; Lugtenberg and Kamilova, 2009). One of the most fascinating topics for research is how plants differentiate between the multitude of “pathogenic” and “useful” microbes living in the rhizosphere. Plant growth promotion *via* PGPR may come about through a variety of ways; for example, regulation of phytohormonal activity, increasing root surface area, and promotion of plant tolerance to diseases (Lucy et al., 2004; Ryu et al., 2005; Dodd et al., 2010; Park et al., 2017), rhizosphere engineering, siderophore production, phosphate solubilization, and the production of active chemical signals (Bhattacharyya and Jha, 2012; Ahemad and Kibret, 2014). These processes have already been observed in plant-PGPR interactions involving important crops such as cereals and legumes (Perez-Montano et al., 2014), canola (Bertrand et al., 2001), tomato (Porcel et al., 2014), pepper (Kokalis-Burelle et al., 2006), and forest trees (Garcia et al., 2004). PGPR have also been used for phytoremediation of contaminated soils (Huang et al., 2004). Furthermore, PGPR-mediated growth promotion has been observed during a variety of stress conditions such as salinity (Lugtenberg and Kamilova, 2009; Karlidag et al., 2013), drought (Lim and Kim, 2013; Vurukonda et al., 2016), heat stress (Meena et al., 2015), metal toxicity (Islam et al., 2014; Khan and Bano, 2016; Bhattacharyya et al., 2017; Khanna et al., 2019), and others.

Genome-guided studies have provided detailed insights into the plant growth promotion potential of various PGPR. Among these, *Bacillus aryabhattai* strains are physiologically versatile (Bhattacharyya et al., 2017) in their plant growth-promoting activities. These strains are known to produce hormones such as IAA, ABA, JA, GAs, and cytokinin in culture (Park et al., 2017), accumulate short-chain-length polyhydroxyalkanoate (Balakrishna Pillai et al., 2017), and produce thermostable proteases (Selim, 2015). Furthermore, *B. aryabhattai* GZ03 is a bagasse-degrading dee seawater strain (Wen et al., 2015). The bacterium appears to be adaptable to a wide variety of ecosystems ranging from deep seawater (Wen et al., 2015) to the upper earth atmosphere (Ramos-Silva et al., 2015). Tibetan plateau environment (Yan et al., 2016). It can be used for heavy metal remediation such as arsenic (Singh et al., 2016), for the production of natural value-added compounds (Paz et al., 2016), and for insecticide degradation (Pailan et al., 2015). However, the plant growth-promoting effects of this bacterial species are limited. Gibberellin production by *Leifsonia soli* is responsible for recovering the dwarf phenotype of GA-deficient Waito-C rice (Kang et al., 2014). For these reasons, PGPR are now widely used as biofertilizers, soil amendments, and rhizoremediators (Kuiper et al., 2004; Bohme and Bohme, 2006; Kalita et al., 2015; Guo et al., 2020). Although the positive effects of PGPR on plant growth have been well-known for decades, the underlying molecular mechanisms of plant growth promotion are poorly understood (Babalola, 2010).

The plant growth-promoting effects of PGPR are mainly derived from morphological and physiological changes of the inoculated plant roots and their functions, and the enhancement of water and mineral uptake (Mohanty et al., 2021). PGPR colonization increases the root surface area as in the case of Azospirillum (Okon and Kapulnik, 1986), promotes cell division of wheat roots (Levanony and Bashan, 1989), increases the diameter and length of lateral roots of maize (Dos Santos et al., 2020), and enhances the development of root hair and cortex (Okon and Kapulnik, 1986).

Some of the most efficient PGPRs are selected based on their root colonizing ability, the time they take to colonize the roots, and the time taken for the bacterial population to reach the log or peak phase (Majeed et al., 2015). For example, *Bacillus amyloliquefaciens* detects root exudates in the soil and successfully colonizes banana roots (Yuan et al., 2015). PGPR are known to produce phytohormones in culture and help the plants maintain a balance between different phytohormones under normal and stress conditions (Park et al., 2017) however, the ability of PGPR to tolerate different stresses has not been well-investigated. Host-PGPR compatibility is another important aspect of PGPR research. Strains compatible with plants from different genera or families are more versatile and in demand. Mixtures of different PGPR, also known as PGPR consortia have also been suggested for use in double or triple cropping systems (Liu et al., 2018; Santoyo et al., 2021).

Furthermore, different metabolites, essential and non-essential amino acids play a major role in maintaining normal physiology (Fukushima, 2011). Similarly, different organic acids in plants are central to metabolism at the cellular level and are released at the root-soil interface to improve mineral acquisition and tolerance against toxic metals (Panchal et al., 2021). Information on PGPR-mediated alterations in plant metabolites, amino acids, and various organic acids is scarce. Against this backdrop, biofertilizers carrying various plant growth-promoting microbial inoculants have been globally accepted as an effective source of synthetic fertilizers being environment-friendly and can be successfully incorporated into different integrated pest/crop management programs. Here we report our findings regarding the plant growth-promoting effects of the rhizobacterium *Bacillus aryabhattai* and the underlying mechanisms.

## Materials and methods

### Isolation and identification of *B*. *aryabhattai*

Bacteria were isolated and identified as described earlier (Park et al., 2017). Briefly, the bacteria were isolated from the rhizospheric soil of soybean plants in the field in Danyang, Chungcheong buk-do, South Korea (Google Map coordinates 36.998671, 128.348711). For this purpose, a suspension of 1 g soil in 9 ml sterile 0.85% NaCl was made and serially diluted up to 10^−6^. Next, 100 μl of the final dilution of the soil suspension was spread on tryptic soy agar (TSA) plates at 30°C for 24hrs. Pure cultures were used for 16s rRNA sequencing. The phylogenetic position of the bacterial isolates was determined by blasting the sequence on NCBI (http://blast.ncbi.nlm.nih.gov/Blast.cgi).

### Brightfield and scanning electron microscopy

For brightfield microscopy, the bacteria were grown on LB-broth at 30°C for 24hrs and 1µl of the culture was used to observe bacteria. Scanning electron microscopy was performed to confirm the successful colonization of the plant roots by the bacteria. Inoculated soybean plants were up-rooted carefully, and freeze-dried after carefully washing with sterile distilled water. The roots were then cut into approximately 5mm^2^ pieces and sputter-coated with gold using an ion sputtering device (JFC-110E, EC&G, USA) and visualized with an FE-SEM scanning electron microscope (S-4300, Hitachi, Japan).

### Rice growth conditions and inoculation with SRB02 cultural filtrate

Seeds of the Gibberellic acid (GA) deficient rice cultivar Waito-C (Mitsunaga and Yamaguchi, 1993; Nishijima et al., 1993) were surface sterilized in 2.5% NaOCl and rinsed with autoclaved distilled water. The seeds were incubated for 24 h with 20 ppm of the GA inhibitor uniconazole (Ikeda et al., 2001). Seeds were plated on 0.8% agar medium in a growth chamber with 14h-28°C/10h-18°C day/night cycle, relative humidity of 60 to 70%, and light intensity of 1000 µmol/m^2^/s for 10 days. When the plants were at the two-leaf stage, 10 µl of SRB02 bacterial culture filtrate was applied near the crown area of the rice seedlings. Data on root and shoot length, chlorophyll content, and fresh and dry weights were recorded after 10 days and compared with distilled water and 0.8% agar media control treatments.

### Soybean growth conditions and inoculation with SRB02

Soybean seeds of the cultivar Daewon were germinated on sterilized soil (fungus-free biosoil, Dongbu Farm, Hannong, South Korea) in plastic pots at 28°C/25°C (day/night), 65% relative humidity, and at a light intensity of 1000 μE/m^2^/s under long-day conditions (16 h light and 8 h dark). The soil of the pots containing soybean plants was drenched with 10ml of 24hrs old LB-grown SRB02 bacterial culture (OD_600_ = 0.02). Control plants were irrigated with 10ml of LB or sterilized distilled water. Plants were inoculated the same way once every day for three days. Following a three-day acclimation period, the plants were kept at 28°C/25°C (day/night), 65% relative humidity, and at a light intensity of 1000 μE/m^2^/s under long-day conditions (16 h light and 8 h dark).

### Data collection on growth parameters

Data were collected on different growth parameters such as root length (RL), shoot length (SL), fresh weight (FW), and dry weight (DW) after 10 days of bacterial inoculation. All data were recorded on 10 plants per replicate and the experiment was repeated 3 times.

### Inductively coupled plasma mass spectrometry analysis

We used inductively coupled plasma mass spectrometry (ICP-MS) to determine the quantity of Ca, Mg, K, P, Fe, Mn, and Na in leaf samples of soybean plants inoculated with the selected bacterial isolate as described by Methela et al. (2023). For this purpose, 0.1g lyophilized leaf powder samples were soaked in 5ml of 70% HCl and put into microwave digestion system (MDS, Milestone, UltraWAVE, USA) for 30min and diluted with 2% HCl. The samples were analyzed using an ICP-MS spectrophotometer (Optima 7900DV ICP-PES, PerkinElmer, USA).

### Amino acid measurements

Extraction of the amino acids was carried out by hydrolyzing 0.05 g of freeze-dried powdered plant sample with 1ml of 6N HCl at 110°C with gentle shaking at 50rpm for 24hrs. The samples were sonicated after mixing 1ml of 0.02N HCl and filtered through a 0.45μm syringe filter (Sterlitech, Auburn, WA, USA). Amino acids were quantified in a High-Speed amino acid analyzer (L-8900, Hitachi, Japan) attached to a HITACHI HPLC system (packed column with ion-exchanging resin; No. 2622 PF; 4.6 mm × 60 mm). Buffer systems used in the mobile phase consisted of Wako L-8500 solutions PF-1, 2, 3, 4, and RG. Measurements were performed using ninhydrin reagent set (Wako Chemical Inc., Japan) by injecting 20 μl samples in replicates.

### 2-Dimensional gel electrophoresis analysis and protein identification

Total protein from soybean leaves was extracted after 24hrs of SRB02 inoculation, in sample lysis solution composed of 7M urea, 2M thiourea with 4%(w/v) 3-[(3-cholamidopropy) dimethyammonio]-1-propanesulfonate (CHAPS), 1%(w/v) dithiothreitol (DTT) and 2%(v/v) pharmalyte and 1mM benzamidine for 1hr at room temperature with frequent vortexing. Samples were then centrifuged at 15,000g for 1hr at 15°C. The soluble portion or supernatant was transferred to a fresh tube and used for two-dimensional gel electrophoresis. Protein concentration was measured *via* Bradford assay (Bradford, 1976). The IPG dry strips (4-10 NL IPG, 24cm, Genomine, Korea) were equilibrated for 12-16hrs with 7M urea, 2M thiourea containing 2% 3-CHAPS, 1% DTT, 1% pharmalyte and loaded with 200µg of the sample. Isoelectric focusing (IEF) was performed at 20°C using a Multiphor II electrophoresis system (Amersham Biosciences) following the manufacturer’s instructions. For IEF, the voltage was linearly increased from 150 to 3,500V during the 3 hours for sample entry followed by constant 3,500V, with focusing complete after 96kVh. The strips were incubated for 10 minutes in equilibration buffer (50mM Tris-Cl, pH6.8 containing 6M urea, 2% SDS, and 30% glycerol), before the second dimension; first with 1% DTT and second with 2.5% iodoacetamide. Equilibrated strips were loaded onto SDS-PAGE gels (20 x 24cm, 10-16%). SDS-PAGE was performed using a Hoefer DALT 2D system (Amersham Biosciences) following the manufacturer’s instructions. 2D gels were run at 20°C and 1,700Vh. The 2D gels were then silver stained as described by Oakley et al. (1980) omitting the fixing and sensitization step with glutaraldehyde. Digitized gel images were quantified using PDQuest (version 7.0, BioRad). The quantity of each spot was normalized *via* total valid spot intensity. Protein spots with significant variation in expression (at least two-fold) compared to the control treatment were selected. Gels were normalized in PDQuest.

Differentially expressed protein spots were identified using MALDI-TOF-MS according to Park et al., 2017. Briefly, the gel spots were digested with trypsin and analyzed using a Voyager-DE STR (matrix-assisted laser desorption ionization time-of-flight) and MALDI-TOF mass spectrometer (PerSeptive Biosystems). The individual protein spots were isolated and re-melted using 93:5:2 water, acetonitrile, and trifluoroacetic acid digestion mixture. The samples were then sonicated for five minutes and centrifuged; 2-μl sample was added to 2-μl peptide sample solution (Kim et al., 2004), and 1 μl of this was placed on the MALDI plate and left for five minutes, after which the samples were washed with 0.1% v/v TFA. Des-Arg1-bradykinin (m/z 904.4681) and angiotensin 1 (m/z 1296.6853) were used as internal standards for calibration. For data analysis, the PerSeptive-Grams software was utilized. Database searches for protein identification were performed using Protein Prospector (http://prospector.ucsf.edu).

### Identification of SRB02 secreted metabolites via GC-MS

Metabolites were identified via GC-MS as described by Park et al. (2021). SRB02 liquid cultures were prepared in 1L and 5L LB at 28°C for 2 days. Cultures were dissolved in methanol and GC analysis was performed using the Agilent 7890B GC (Agilent, USA) system coupled with Agilent MSD 5977B quadruple mass spectrometer (Agilent, USA) and capillary column of 60m length, 0.25 µm i.d., 0.25 µm film thickness (DB-WAX, Agilent, USA). A 10 µl of the sample was injected with an initial oven temperature of 40°C for 2 min followed by a gradual increase to 220°C @ 3°C/min and held at 220°C for 10 min. Helium was used as the carrier gas at a constant flow rate of 1mL/min. A series of n alkanes (C6-C22) was analyzed under the same conditions to obtain the Linear Retention Index values for the reaction mixture components. Electron ionization mass spectra in the range of 35–350 m/z were recorded at 70 eV and an emission current of 50 µA and scanned from m/z 28 to 450 at 1.7 scans/s. The mass spectra obtained were compared with the Wiley 275 mass spectral database (Agilent, USA).

### Treatment of plants with butanoic acid

**Figure 1:**
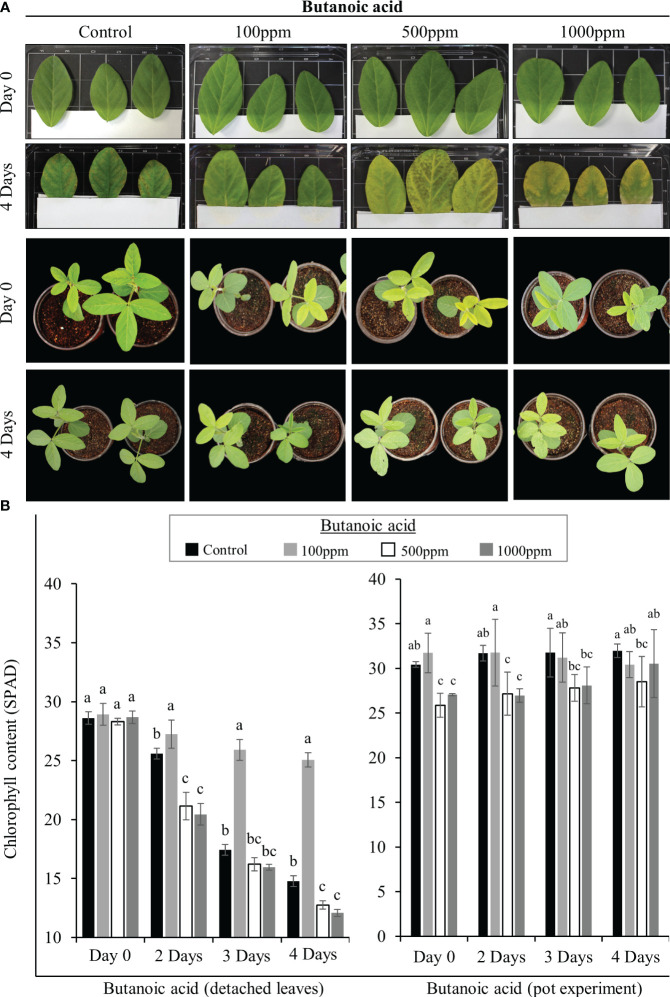
SRB02-mediated butanoic acid (BA) production and chlorophyll preservation. Detached soybean leaves were treated with 100, 500, and 1000ppm of butanoic acid and data were recorded on the 4th day. BA in its lowest concentration of 100ppm appeared to preserve the chlorophyll content after 4 days of its application to detached leaves. A similar experiment was performed on pot-grown live plants with almost the same result **(A)**. The 100ppm BA appeared to maintain or preserve the chlorophyll content in the detached leaves as well as the leaves of pot-grown plants **(B)**. However, leaves treated with 500ppm and 1000ppm butanoic acid turned yellow indicating that BA is beneficial for plants in low concentrations and that higher concentrations of butanoic may be harmful for soybean plants. All data points show an average of at least three replicates. Error bars show standard deviation. The small letters indicate significant differences between the means.

For the “tissue plate” assay (Mun et al., 2021), at least three leaves from soybean plants (V3 stage) were detached and surface sterilized. Next, sterilized tissue papers were kept in 12cm square petri plates covering the bottom half of the plate. The detached leaves were placed on top of the tissue paper such that the leaf petiole side was on top of the tissue paper. The leaves were then sandwiched between sterilized tissue papers as shown in **Figure 1A**. Finally, 10ml of 100ppm, 500ppm, and 1000ppm concentrations of butanoic acid were poured on the tissue paper and the plates were covered with lids and incubated at 30/25°C (day/night) temperature for 5 days.

Next, we performed the same type of experiments on live soybean plants grown in soil. For this purpose, plants at the V3 stage were used. The same three concentrations of butanoic acid in a 10ml solution were poured into the soil. Phenotypic observations were recorded every day and chlorophyll measurements were conducted after 2, 3 and 4 days of the treatment.

### Gene expression analysis

Total RNA was extracted using Trizol reagent (Invitrogen, USA) followed by ethanol precipitation before cDNA synthesis. Following the necessary quality control checks, 1µg RNA was used to synthesize cDNA using a cDNA synthesis Kit (PhileKorea, South Korea) according to the manufacturer’s instructions. Quantitative RT-PCR was performed in Eco™ real-time PCR machine (Illumina, USA), using a 2x Quantispeed SYBR Mix (PhileKorea, Korea) with 10nM of each primer (**Supplementary Table S1**) and 100 ng of template DNA, in a final volume of 20μl in a two-step PCR reaction for 40 cycles under the following conditions: Initial denaturation and polymerase activation at 95°C for 2min and subsequent denaturation steps at 95°C for 10s followed by annealing and extension at 60°C for 30s.

### Statistical analysis

Laboratory plate-based experiments were set up in a completely randomized design (CRD) whereas the greenhouse experiments were conducted in a randomized complete block design (RCBD). Experiments were performed at least three times. Data were collected in triplicate and subjected to ANOVA and the Duncan multiple range test using the SAS version 9.2. Furthermore, the data were graphically presented using Microsoft Excel or GraphPad Prism for Windows (GraphPad Software, San Diego, California USA, www.graphpad.com).

## Results

### Identification of *Bacillus aryabhattai* SRB02 as PGPR

Brightfield/light and scanning electron microscopic observations showed 0.8 to 1.5µm long rod-shaped Bacilli (**Supplementary Figures S1A, B**). Based on the 16s rRNA sequence, the bacterium was identified as *Bacillus aryabhattai* by blasting the sequence on NCBI [BLAST: Basic Local Alignment Search Tool (nih.gov)]. Phylogenetic analysis indicated 100% similarity to the *B. aryabhattai* strain SRB02 [(GenBank: KP860638.1) *Bacillus aryabhattai* strain SRB02 16S ribosomal RNA gene, partial sequ - Nucleotide - NCBI (nih.gov)]. Plant growth-promoting rhizobacteria are beneficial for their host plants in several ways. A physical association or contact of the rhizobacteria with the roots greatly enhances the chances of obtaining maximum benefits of the association for both organisms. Scanning electron microscopy of cleaned and washed roots of both the rice and soybean plants showed colonization of the roots by SRB02 in soil within 2 days of the inoculation (**Supplementary Figure S1B**).

### *Bacillus aryabhattai* SRB02 promotes the growth of GA-deficient rice and soybean plants

**Figure 2:**
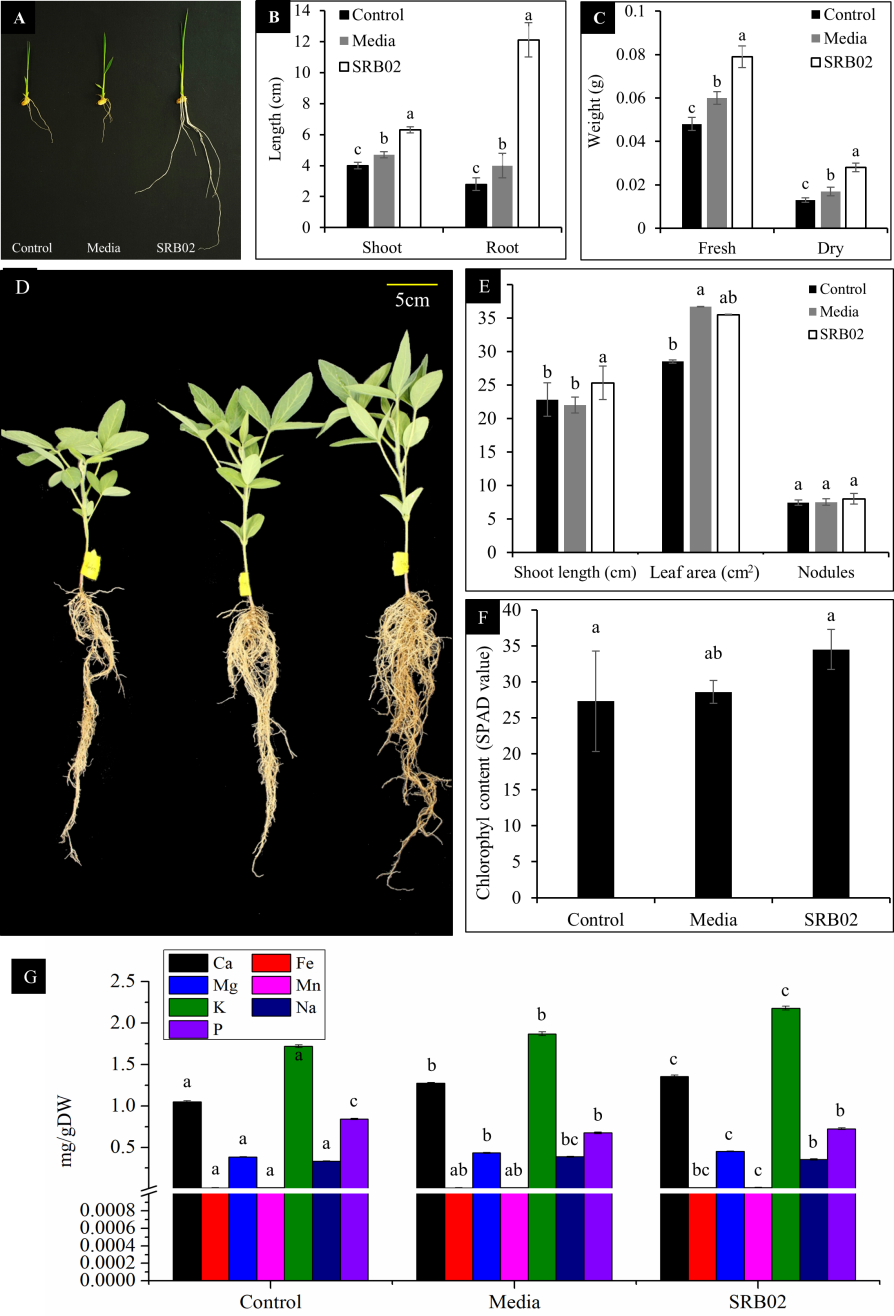
*Bacillus aryabhattai* SRB02 promotes the growth of GA-deficient rice plants. *B. aryabhattai* SRB02 promoted the growth of GA-deficient Waito-C rice seedlings **(A)** with at least six times longer shoots and roots than that of the control plants **(B)** as well as significantly higher biomass as reflected by fresh and dry weights **(C)**. *B. aryabhattai* SRB02 inoculation promoted the growth of soybean plants **(D)** with statistically higher shoot length and leaf area as compared to the untreated plants **(E)**. Furthermore, the leaves of the SRB02-treated plants had significantly higher chlorophyll content **(F)**. ICP analysis indicated a significantly higher accumulation of Ca, Mg, and K in the leaves of SRB02-treated plants **(G)**. All data points show an average of at least three replicates. Bars with different letters indicate significant differences. Error bars show standard deviation.

We inoculated SRB02 to the seedlings of the GA-deficient rice cultivar Waito-C and observed a significantly positive impact of SRB02 on the overall growth of these plants as compared to the control plants which were treated with sterilized distilled water and LB media only (**Figure 2A**). The SRB02-treated rice plants had significantly longer shoots and roots (more than 5 times) than that of the control plants (**Figure 2B**) as well as higher biomass as reflected by the statistically higher fresh and dry weights of these plants (**Figure 2C**).

SRB02 significantly promoted the growth of soybean plants as shown by statistically higher shoot lengths and root biomass as compared to the WT plants (**Figures 2D, E**). Though no statistical difference in the number of root nodules was observed, the leaf area of SRB02-treated plants was statistically higher than that of the WT plants (**Figure 2E**) along with a significantly higher chlorophyll content (**Figure 2F**). ICP analysis of the plants indicated a significantly higher accumulation of Ca, Mg, and K in the SRB02-treated plants (**Figure 2G**).

### *Bacillus aryabhattai* increases amino acid content in soybean

To further investigate the impact of SRB02-mediated increase in plant biomass and different ions, we determined the quantity of 18 different amino acids in the leaves of soybean plants *via* HPLC. Results indicated a statistically significant increase in the quantity of different amino acids in the SRB02-treated plants. The amino acids threonine, serine, glycine, tyrosine, lysine, histidine, and arginine increased by 99, 146, 104, 103, 80, 91, and 98 per cent, respectively compared to the leaves of the control plants (**Table 1**). Furthermore, most of the other amino acids exhibited an increase of more than 50% in the PGPR SRB02-treated plants (**Table 1**).

**Table 1:** Comparison of the leaf amino acid composition of SRB02-treated pot-grown soybean plants with the control plants.

S. No.	Amino acid	Control	Media	SRB02	Increase over control (%)
		Quantity in leaves (mg/g DW).			
1	Aspartic acid	3.198 ± 0.66 a	5.870 ± 0.40 b	5.791 ± 0.44 b	81.082
2	Threonine	1.319 ± 0.21 a	2.324 ± 0.57 b	2.629 ± 0.55 b	99.318
3	Serine	0.814 ± 0.39 a	1.839 ± 0.33 b	2.007 ± 0.70 b	146.560
4	Glutamic acid	2.523 ± 0.49 a	3.174 ± 0.52 ab	3.365 ± 0.19 b	33.373
5	Glycine	0.428 ± 0.05 a	0.681 ± 0.14 b	0.875 ± 0.39 bc	104.439
6	Alanine	3.217 ± 0. 22 a	4.703 ± 0.39 b	5.391 ± 0.20 c	67.578
7	Cystine	0.525 ± 0.21 a	0.854 ± 0.32 ab	0.993 ± 0.17 b	89.143
8	Valine	2.945 ± 0.52 a	3.864 ± 0.43 b	4.178 ± 0.61 bc	41.868
9	Methionine	0.320 ± 0.16 a	0.242 ± 0.05 b	0.335 ± 0.06 a	4.688
10	Isoleucine	2.690 ± 0.37 a	3.804 ± 0.34 b	4.424 ± 0.61 bc	64.461
11	Leucine	3.847 ± 0.66 a	5.510 ± 0.52 b	6.535 ± 0.41 c	69.873
12	Tyrosine	1.032 ± 0.21 a	1.813 ± 0.33 b	2.099 ± 0.31 b	103.391
13	Phenylalanine	2.127 ± 0.32 a	3.087 ± 0.41 b	3.700 ± 0.29 c	73.954
14	Lysine	1.341 ± 0.46 a	2.060 ± 0. 34 ab	2.548 ± 0.26 b	90.007
15	Ammonia	0.787 ± 0.11 a	1.001 ± 0.25 ab	1.046 ± 0.13 ab	32.910
16	Histidine	0.851 ± 0.19 a	1.413 ± 0. 24 b	1.633 ± 0.20 b	91.892
17	Arginine	2.122 ± 0.45 a	3.616 ± 0.43 b	4.202 ± 0.18 c	98.021
18	Proline	2.719 ± 0.68 a	3.909 ± 0. 33 b	4.397 ± 0.24 c	61.714
**Total amino acid content**		**32.806**	**49.763**	**56.149**	**1354.271**

Values followed by different letters are statistically different at p≤0.05.

### Identification of PGPR SRB02-responsive proteins *via* 2-DE gel analysis

**Figure 3:**
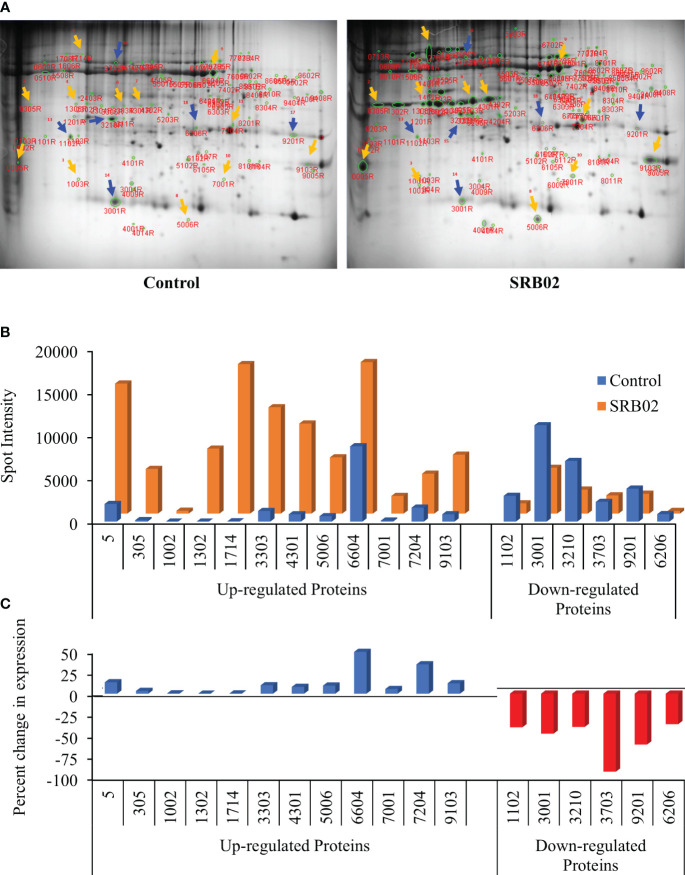
Identification of *B. aryabhattai* SRB02-responsive proteins via 2-DE gel analysis. Results of the 2-DE gel assay identified several differentially accumulated proteins with significant differences in the number, intensity, and position of protein spots on the two gels **(A)**. The accumulation of 12 proteins was found to be significantly increased following SRB02 application but the expression of 6 genes was reduced **(B)**. Furthermore, the accumulation of certain proteins increased by up to 50%, whereas others increased by up to 34%. On the other hand, the decrease in the accumulation of some proteins ranged between 90% and 30%, following the SRB02 application **(C)**.

PGPR-mediated enhancement of plant growth regulation occurs due to a variety of reasons. However, at the cellular level, it is manifested in the form of significant changes in the transcription and translation of key genes involved in important physiological pathways leading to enhanced plant growth. The transcriptional and translational patterns of a gene are important for understanding its role in a particular pathway and getting a complete picture of the gene’s function. Hence, the characterization of proteins with significant differences before and after the application of PGPR will lead to the identification of key genes regulating PGPR-mediated plant growth and development. Therefore, we performed a 2-dimensional electrophoretic (2-DE) gel assay to isolate proteins that are differentially expressed in SRB02-treated plants after 24hrs. The 2-DE separates proteins based on two properties in two dimensions and is suited to separate diverse proteins. Here we detected significantly diverse proteins spread across the gel. Significant differences were observed in the number, intensity, and position of protein spots on the two gels corresponding to proteins extracted from control and SRB02-treated soybean leaves (**Figure 3A**). For further analysis, we selected 18 differentially expressed protein spots. Among these, the expression of 12 proteins was found to be significantly increased following SRB02 application whereas, the expression of 6 genes was reduced (**Figure 3B**). Following the SRB02 application, the accumulation of certain proteins increased up to 50%, whereas others increased by up to 34%, 13%, and some up to less than 1%. However, the accumulation of some proteins was reduced by more than 90%, 60%, 73%, and 30%, following SRB02 application (**Figure 3C**).

### Identification of differentially expressed proteins and metabolites via MALDI-TOF and GCM-MS

Differentially expressed protein spots were identified using MALDI-TOF-MS as described earlier (Park et al., 2017). The identified proteins included Glyma13g07616 (corresponding to spot 1714) and Glyma07g01730 (corresponding to spot 7204) with 137- and 2.8-fold increase in their accumulation, respectively, following SRB02 application (**Table 2**). These genes encode β-conglycinin and a putative uncharacterized protein belonging to the HAD superfamily, respectively. Interestingly, two other over-accumulated glycinin proteins (spots 3303 and 4301) were also found, but the identity of the genes encoding these proteins could not be established. The accumulation of these proteins was 10 and 12.4 folds higher than in the control gel. Similarly, the protein spot 6604 was identified as a large subunit of the ribulose-1,5-bisphosphate carboxylase/oxygenase (NCBI: gi|91214125) over-accumulated by 2.01 folds following SRB02 application. The protein spot number 5 was identified as Glyma08g341500 encoding a Kunitz trypsin inhibitor involved in modulating programmed cell death during infection, over-accumulated by 7.3 folds. Genes encoding proteins with a decrease in their accumulation in response to PGPR application were also identified (**Table 2**).

**Table 2:** Identification of 2D Gel spots *via* QTOF-LCMS.

S. No.	Spot No.	NCBI Accession	SoyBase Gene ID	Description	Expression	Fold change
1	5	gi|157838209	Glyma08g341500	Soybean trypsin Inhibitor. Encodes a Kunitz trypsin inhibitor involved in modulating programmed cell death in plant-pathogen interactions.	Increased	7.39
2	1102	gi|359806638	Glyma08g082900	Chlorophyll a-b binding protein 3, chloroplastic. Heat stress-induced response of the proteomes of leaves from *Salvia splendens* Vista and King	Decreased	0.40
3	1714	gi|335353923	Glyma20g148300	Beta-conglycinin alpha subunit	Increased	137.02
4	3001	gi|351724437	Glyma13g07610	Ribulose-1,5-bisphosphate carboxylase small subunit rbcS2	Decreased	0.47
5	3210	gi|356559442	Glyma16g143600	Oxygen-evolving enhancer protein 1, chloroplastic-like. Leaf Proteome Analysis Reveals Prospective Drought and Heat Stress Response Mechanisms in Soybean	Decreased	0.39
6	3303	gi|254029113	–	Mutant glycinin subunit A1aB1b	Increased	10.02
7	4301	gi|254029115	–	Mutant glycinin subunit A1aB1bsp	Increased	12.41
8	5006	gi|947103608	Glyma06g039700	Hypothetical protein GLYMA_06G039700	Increased	10.46
9	6604	gi|91214125	–	Ribulose-1,5-bisphosphate carboxylase/oxygenase large subunit	Increased	2.01
10	7001	gi|351722245	Glyma.02g076600	Seed maturation protein PM31 Heat stress effects on ribulose-1,5-bisphosphate carboxylase/oxygenase, Rubisco binding protein and Rubisco activase in wheat leaves	Increased	18.04
11	7204	gi|255646711	Glyma07g01730	Unknown (Putative uncharacterized protein) or stem 28 kDa glycoprotein precursor. HAD superfamily, subfamily IIIB acid phosphatase	Increased	2.86
12	3703	gi|91214125	Glyma.12g061600	Raibulose-1,5-bisphosphate carboxylase/oxygenase large subunit	Increased	0.92
13	9103	gi|15988118	Glyma03g32030	Chain A, crystal structure of soybean Proglycinin A1ab1b homotrimer	Increased	11.62
14	9201	gi|351727983	Glyma07g014500	Stem 28 kDa glycoprotein precursor	Increased	0.60

As described earlier, PGPR-mediated plant growth promotion occurs due to different reasons. These include the production of important metabolites which are then secreted and absorbed by the plants subsequently contributing to plant growth, development, and immunity. Therefore, we analyzed the SRB02 culture filtrates for the presence of different metabolites via GCMS analysis. We performed the analysis for 1L and 5L cultures assuming that a larger volume of culture may provide a chance to identify a higher number of metabolites and a much deeper understanding of metabolite production by PGPR. Interestingly, significantly higher quantities of butanoic acid and 3-methyl butanoic acid were found in the cultural filtrates (**Table 3**). The quantity of butanoic acid was about 42% and 24% of the identified metabolites in 1L and 5L cultures, respectively. This led us to suspect that butanoic acid may be a major contributor to the plant growth-promoting abilities of SRB02.

**Table 3:** GC-MS- based identification of different metabolites in the SRB02 liquid culture.

Culture	Peak Number	Retention Time	Area Percentage	Library/ID
**1L**	10	21.5003	11.9513	Acetic acid
	14	23.7885	4.6783	Propanoic acid, 2-methyl-
	15	24.8521	4.4128	Butanoic acid
	17	25.5267	24.7142	Butanoic acid, 3-methyl-
	34	37.8408	3.6513	Acetic acid, phenyl-
	38	44.7397	10.2957	1,3-Cyclohexanedione, 2,5,5-trimethyl-
	40	45.5479	8.9985	Diethyltrisulphide
**5L**	12	18.5128	1.0315	Pyrazine, 2,5-dimethyl-
	18	21.4644	4.6633	Acetic acid
	22	23.2233	1.8679	Propanoic acid
	25	23.7302	5.1929	Propanoic acid, 2-methyl-
	26	24.833	2.5418	Butanoic acid
	29	25.4534	13.0896	Butanoic acid, 2-methyl-
	66	34.6346	4.7491	Propanoic acid, 3-(methylthio)-
	78	37.8155	5.5631	Acetic acid, phenyl-
	79	38.3488	2.197	DL-Proline, 5-oxo-, methyl ester
	84	39.8216	1.0641	3-Pyridinecarboxylic acid
	101	43.4304	5.4146	1,3-Cyclohexanedione, 2,5,5-trimethyl-
	105	44.7962	6.333	Diethyldithiophosphinic acid
	106	44.9269	1.0896	Hydrindacen-1-ene
	107	45.6078	5.8156	Diethyltrisulphide
	108	45.9203	2.9577	(2S,9S) 9-Isopropyl-1,7-dioxo-2,8-diazabicyclo [4.3.0] nonane

### SRB02-mediated butanoic acid production and chlorophyll preservation

After detecting significantly higher quantities of butanoic acid in SRB02 culture filtrates, we tested different concentrations of commercial butanoic acid to determine its role in plant growth promotion. For this purpose, we treated detached soybean leaves and treated them with 100, 500, and 1000ppm of butanoic acid (also known as isovaleric acid) as described by Mun et al. (2021) and recorded our observations on the 4^th^ day. Butanoic acid in its lowest concentration of 100ppm appeared to preserve the chlorophyll content after 4 days of its application to detached leaves as well as live plants (**Figure 1**). Detached leaves in the control treatment (water only) developed necrotic symptoms after 4 days. However, detached leaves treated with 100ppm butanoic acid remained fresh and green indicating that the chlorophyll is still intact (**Figure 1A**). Leaves treated with 500ppm and 1000ppm butanoic acid lost their chlorophyll and became chlorotic after 4 days. Next, we performed the same type of experiments on live plants grown in pots as suspected that the chlorosis observed in the earlier experiment may be due to the detachment of the leaves from the plants. However, we observed the same effect of butanoic acid on live soybean plants. After 4 days, plants treated with 100ppm butanoic acid were dark green, whereas those treated with 500ppm and 1000ppm butanoic acid turned yellow (**Figure 1A**). These results were supported by the chlorophyll measurements in the detached leaves from the “tissue plate” experiment as well as leaves from the pot-grown plants. Significantly higher chlorophyll contents were recorded in leaves after 2, 3, and 4 days of treatment with 100ppm butanoic acid (**Figure 1B**). Taken together, these results indicate that butanoic acid is beneficial for plants only in low concentrations and higher concentrations of butanoic acid are rather harmful for soybean plants.

**Figure 4:**
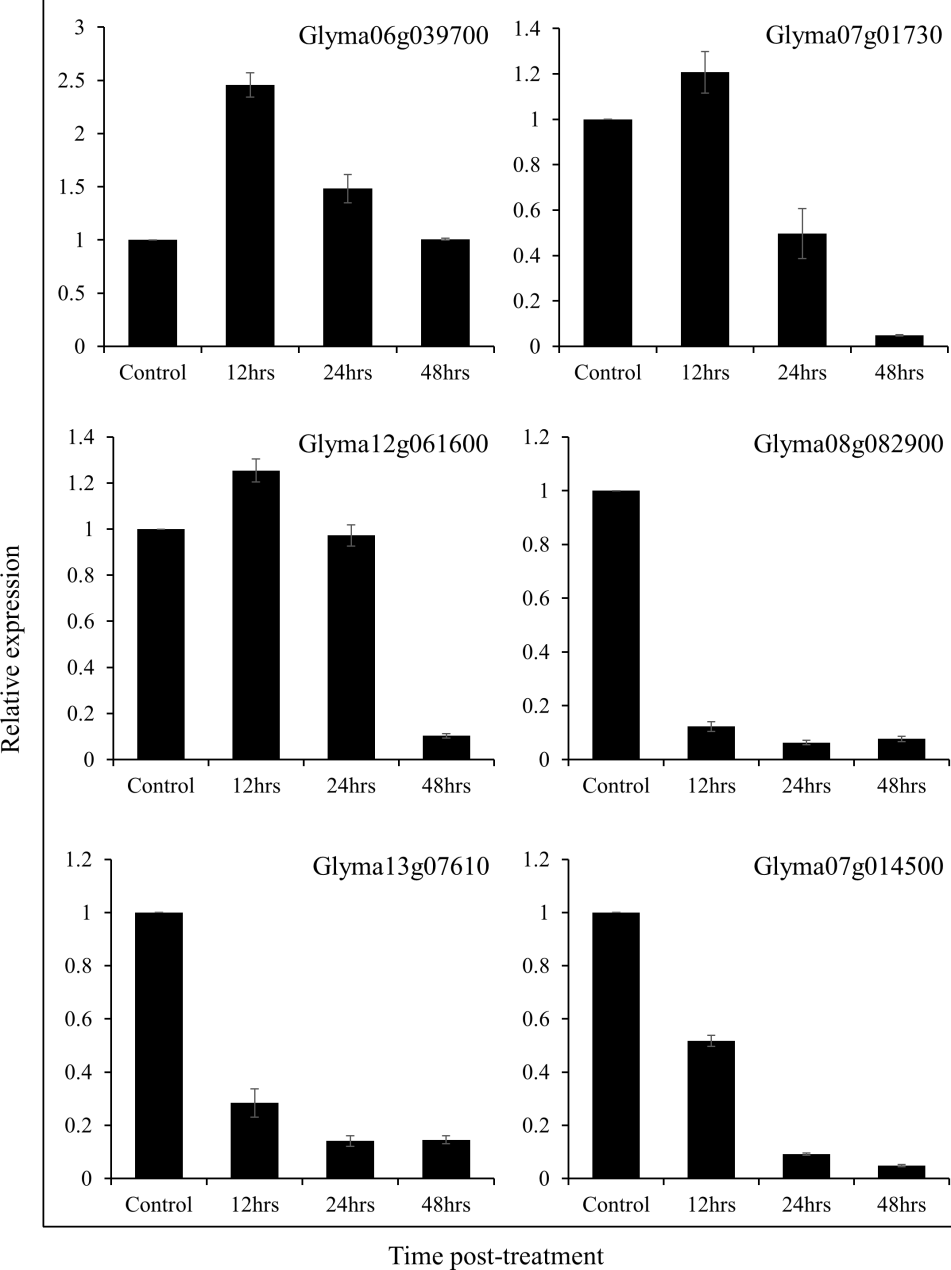
Real-time quantitative PCR analysis. The results of the real-time QPCR analysis were consistent with the protein expression patterns observed in 2DE gels. A significant increase in the expression of Glyma06g039700, Glyma07g01730, and Glyma02g061600 in soybean leaves was recorded after 12 hrs of treatment with SRB02. Whereas, the expression of Glyma08g082900, Glyma13g07610, and Glyma07g014500 was significantly reduced. Each data point represents the mean of at least three replications and the error bars represent standard deviation.

Real-time quantitative PCR analysis of multiple genes was consistent with the protein expression patterns observed during the 2DE gel analysis. The expression of Glyma06g039700, Glyma07g01730, and Glyma02g061600 increased significantly in soybean plants after 12 hrs of treatment with SRB02 but dropped back after 24 and 48 hours (**Figure 4**). On the other hand, the expression of Glyma08g082900, Glyma13g07610, and Glyma07g014500 was continuously reduced after 12, 24 and 48 hours of SRB02 application (**Figure 4**).

## Discussion

The use of plant growth-promoting rhizobacteria (PGPR) as microbial inoculants is becoming increasingly popular in intensive agriculture. Efficient PGPR are selected based on their ability to aggressively colonize plant roots. Various PGPR have been reported to colonize roots within one to several weeks, with bacterial populations peaking within 60 days (Majeed et al., 2015). *Bacillus aryabhattai* strain SRB02 identified in this study successfully colonized soybean roots within 2 days of inoculation. *Bacillus amyloliquefaciens* (NJN-6) is another PGPR from the same genus that can detect root exudates in the soil and successfully colonize banana roots (Yuan et al., 2015). We have previously characterized this PGPR as *Bacillus aryabhattai* SRB02 via 16s rRNA sequencing (Park et al., 2017). The bacterial strain has an amazing potential to be used as a PGPR owing to its versatile properties. For example, the bacterium does not only produce several phytohormones such as ABA, IAA, JA, GA and CK in culture, but it also helps the plants maintain a balanced phytohormone production under normal as well as under stress conditions (Park et al., 2017). A good deal of research has been conducted on the use of PGPR for promoting plant growth under stress where different plant species are subjected to biotic and/or abiotic stresses. However, studies, where the PGPR have been tested for their ability to efficiently work under stress, are no more than a handful. In an earlier study, we have also tested the ability of SRB02 to tolerate significantly higher levels of oxidative and nitrosative stress conditions which we think is a highly important trait required for the field applicability of SRB02 (Park et al., 2017). The magnitude of oxidative and nitrosative stress treatments we have used for this purpose is enough to even completely suppress the seed germination of different plant species in our laboratory. Under field conditions, plant growth-promoting bacteria are exposed to a variety of environmental stresses, therefore, PGPR needs to be able to survive under stress and SRB02 qualifies this criterion with good confidence.

The compatibility of a PGPR with multiple plant species is an added advantage. *Bacillus aryabhattai* SRB02 is compatible with monocotyledonous rice as well as dicotyledonous soybean plants. Mixtures of different PGPR have been suggested for use in multiple cropping systems to ensure the availability of PGPR for every type of plant host (Liu et al., 2018). However, different PGPRs in a single mixture may inhibit each other due to possible cross-talk or the production of inhibitory compounds. Furthermore, the diminishing efficacy of different PGPR as bioinoculants has been known due to strong competition for rhizosphere colonization (Santoyo et al., 2021). Therefore, the compatibility of a single PGPR with multiple plant hosts is a plus point.

The Waito-C is a mutant line of rice in which the biosynthetic pathway of gibberellins is genetically blocked (Mitsunaga and Yamaguchi, 1993) resulting in a significantly dwarf phenotype. SRB02 application to Waito-C plants resulted in a normal or wild-type phenotype with increased plant height and root length. We have previously shown that SRB02 not only produces significant amounts of GA derivatives in culture but also promotes GA production in the host plants under normal as well as stress conditions (Park et al., 2017). In 2014, Kang et al. showed that gibberellin production by the PGPR *Leifsonia soli* strain SE134 was responsible for recovering the dwarf phenotype of Waito-C rice Kang et al. (2014). *Serratia glossinae* strain GS2 also promoted the growth of Waito-C rice plants along with the production of different gibberellins (Jung et al., 2017). Similarly, SRB02 also promoted the growth of soybean plants. No statistical difference between the number of root nodules was expected as the plants were grown in pots and according to our observations, pot-grown legumes usually do not produce nodules especially if they are grown in sterilized soil. Both the rice and soybean SRB02-treated plants had more chlorophyll content in their leaves compared to the control plants. This was accompanied by a concomitant increase in Ca, Mg, and K ions in the SRB02-treated plants. Although Ca, Mg, and K are micronutrients, they are essential mineral nutrients required by plants in relatively larger amounts to execute normal physiological processes including photosynthesis. Potassium and magnesium are rate-limiting factors involved in the process of photosynthesis, long-distance translocation of photoassimilates, and photoprotection (Tränkner et al., 2018). Deficiency or absence of K and/or Mg results in a complete photosynthetic meltdown. Consequently, excessive production of reactive oxygen species (ROS) results in photo-oxidation of the chlorophyll and other photosynthetic machinery along with abnormal phloem loading and long-distance transport of assimilates [for details, see Tränkner et al. (2018)]. Though the role of Ca in photosynthesis is seldom investigated, an armada of calcium-binding proteins in plants are located in the chloroplast or the chloroplast membrane and involved in the process of photosynthesis either directly or indirectly [for details, see Wang et al. (2019)]. Additionally, intracellular Na accumulation disturbs the normal K:Na ratio, ultimately affecting photosynthesis (Sudhir and Murthy, 2004). Interestingly, SRB02 application resulted in a mild reduction in Na ions in soybean leaves. This potentiates that SRB02-mediated improvement in chlorophyll accumulation in this study is associated with the significantly higher quantities of Ca, K, Mg and a mild reduction of Na observed during the ICP analysis.

PGPR-mediated increase in biomass was accompanied by a parallel increase in the quantity of various amino acids in soybean leaves. Some amino acids increased by as much as 146 percent whereas others increased by more than 100 percent following SRB02 application. Different metabolites including amino acids play a critical role in the maintenance of normal physiology under basal as well as induced conditions. However, information on the comparative analysis of various physiological traits and amino acid profiling affected by different PGPR is scarce.

Amino acids, asparagine, aspartate, Vitamins, riboflavin, nicotinamide, and other compounds including glycerol, 4-hydroxy-L-phenylglycine, and 3-hydroxy-3-methylglutarate showed increased levels of accumulation in the leaves of plants treated with PGPR and plant growth regulators (PGR). Khan et al. (2019) reported a significant increase in the amino acid (threonine, methionine, and histidine) content in chickpea plants after the application of a consortium of three different PGPR and plant growth regulators (PGRs). This study provides a comprehensive account of the amino acid profile in response to the lone PGPR *Bacillus aryabhattai* treatment in soybean leaves.

PGPR-mediated enhancement of plant growth regulation manifests in the form of significant variations in the transcription and translation of key genes. Therefore, the identification of differentially expressed proteins before and after the PGPR application will provide a deeper understanding of the molecular mechanisms regulating PGPR-mediated plant growth and development. We found several differentially expressed proteins via 2-DE gel assay. May proteins were upregulated by up to 50% whereas a few proteins were down-regulated by up to 90% after SRB02 application. Interestingly, these included four genes; Glyma03g32030 (spot 13) and Glyma20g148300 (spot 1714) encoding proglycinin A1ab1b homotrimer and β-conglycinin along with two unidentified proteins encoding glycinin subunit A1ab1b and A1aB1bsp, respectively. Glycinin and β-conglycinin are the two major soybean storage proteins. According to careful estimates, approximately 90% of soybean proteins exist as storage proteins mostly consisting of β-conglycinin and glycinin (Fukushima, 2011). Of these, the β-conglycinin is the vicilin (legume globulin storage protein that protects against pathogens) storage trimeric protein of soybeans, composed of three peptides; α, α´ and β. Generally, oil makes up about 19% of the soybean dry weight whereas the storage proteins make up about 40% of the dry seed weight mostly consisting of β-conglycinin (Liu et al., 2022). The significant increase in glycinin and β-conglycinin encoding proteins provides the mechanistic details of SRB02-mediated plant growth promotion in soybean. We performed our analysis on the leaves of soybean plants at the V3 stage, it is safe to assume that the significant increase in the expression of the above proteins in the leaves would ensure a much higher accumulation of the glycinin and β-conglycinin storage proteins in the seeds and may ultimately affect the oil content.

Different organic acids are central to cellular metabolism under basal as well as induced conditions. Several plants are known to exude different organic acids at the root-soil interface that may lead to improved mineral acquisition and toxic metal tolerance (Panchal et al., 2021). Butyric acid (BA) or butanoic acid (also known as isovaleric acid – IVA) is not directly related to plant growth. However, the derivative Indole-3-butyric acid (IBA) is a growth regulator found in plants. It is used on many crops and ornamentals to promote growth and development of roots, flowers and fruits, and to increase crop yields (Ludwig-Müller, 2000). Here, we recorded an increased accumulation of butanoic acid in soybean leaves due to SRB02 inoculation. This increase in BA accumulation may further contribute to the production and accumulation of IBA (a form of auxin) phytohormone as has been reported for SRB02 earlier (Park et al., 2017). The 3-methylbutanoic acid, also known as β-methylbutyric acid or simply butanoic acid is classified as a short-chain fatty acid with an unpleasant odor and is not only present in mammals including humans but is also found in several foods such as apple juice, cheese, and soymilk. Short-chain fatty acids (SCFAs) have a variety of health effects as they are known to regulate lipid and glucose metabolism (Chen et al., 2022). Feeding mice with soybean isoflavones reduced BA accumulation (Huang et al., 2022). Furthermore, Hong et al. (2017) reported that the fermentation of soybean by *Bacillus subtilis* increased the content of BA. More recently, quantitative analysis for several nutrients and volatile components during fermentation of soybean by *Bacillus subtilis* increased a significant and consistent increase in the accumulation of BA starting from 24hrs to 96hrs (last data collection point in the study. As *B. subtilis* is a well-known PGPR with several commercial field-applicable products available in the market, it appears that plant growth promotion via PGPR often involves increase in the accumulation of butanoic acid in the plants. However, butanoic acid being as high as 42% fraction of all the identified metabolites has not been reported elsewhere. Our further investigations involving BA indicated that it helps in preserving or maintain the chlorophyll content in the leaves. How BA helps maintain chlorophyll content is not clearly understood, and information on the exact roles of BA in plant development is largely unknown. Surprisingly however, Murata et al. (2022) recently reported that the volatile butanoic acid from *Bacillus atrophaeus* (ATCC9372) inhibited plant growth in *Arabidopsis thaliana*. Although *B. atrophaeus* is a well-known PGPR, this study only involved the analysis of the volatile compounds released by *B. atrophaeus* cultures and there was no physical contact between the bacteria and the plants.

As the need to produce more from the same piece of land in a much less time has increased significantly, the use of PGPR has now become an essential part of the intensive agriculture system. The PGPR SRB02 used in this study is of particular importance since it not only produces several phytohormones *per se*, but it also contributes to the maintenance of phytohormones, chlorophyll, amino acids, and other metabolites in plants. Furthermore, SRB02 can tolerate oxidative and nitrosative stresses (Park et al., 2017), significantly increasing their field applicability in the form of a biofertilizer.

## Conclusions

PGPR-based microbial inoculants are becoming increasingly popular in intensive agriculture. *Bacillus aryabhattai* SRB02 successfully colonizes soybean roots rapidly and has a great potential to promote plant growth via production of phytohormones, promoting nutrient accumulation and chlorphyl stability.

## Data availability statement

The datasets presented in this study can be found in online repositories. The names of the repository/repositories and accession number(s) can be found below: https://www.ncbi.nlm.nih.gov/nuccore/KP860638.1/.

## Author contributions

B-GM: Conceptualization, Data curation, Investigation, Writing – review & editing. AH: Formal analysis, Software, Supervision, Validation, Visualization, Writing – original draft, Writing – review & editing. Y-GP: Data curation, Investigation, Methodology, Writing – review & editing. SK: Methodology, Project administration, Writing – review & editing. IL: Project administration, Resources, Validation, Writing – review & editing. BY: Conceptualization, Funding acquisition, Project administration, Resources, Supervision, Validation, Writing – review & editing.
