# Supplementary figures and images for: The PGPR Bacillus aryabhattai promotes soybean growth via nutrient and chlorophyll maintenance and the production of butanoic acid
Source: Front Plant Sci. 2024 Feb 19;15:1341993. doi: 10.3389/fpls.2024.1341993 (PMC10909845; doi:10.3389/fpls.2024.1341993)

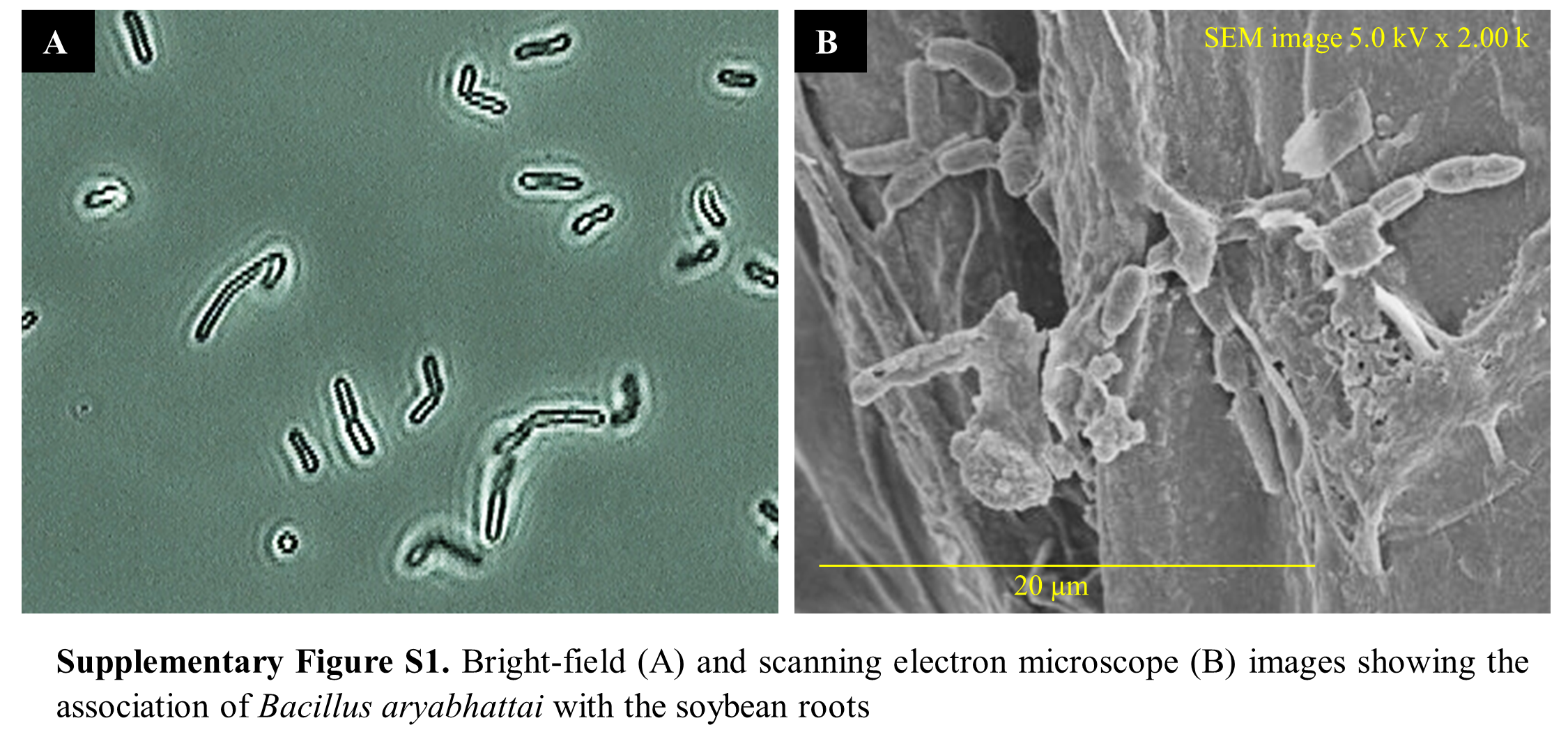

Supplement: Supplementary file 1 [file Image_1.tif]
