# Supplementary material for: The PGPR Bacillus aryabhattai promotes soybean growth via nutrient and chlorophyll maintenance and the production of butanoic acid
Source: Front Plant Sci. 2024 Feb 19;15:1341993. doi: 10.3389/fpls.2024.1341993 (PMC10909845; doi:10.3389/fpls.2024.1341993)
Supplement: Supplementary file 2 [file Table_1.docx]

**Table: List of primers and sequences**

| **S. No.** | **Gene** | **Primer** | **Primer Sequence (5´-3´)** | **Product size (bp)** |
| --- | --- | --- | --- | --- |
| 1 | Glyma.06g039700 | Forward | TGGACTTGCAGAGGATCCGGCA | 428 |
|  |  | Reverse | CCACCATTGCGAGTCGCCCA |  |
| 2 | Glyma07g01730 | Forward | ACAAGGGCAATGCACCGGCA | 298 |
|  |  | Reverse | TGGCCTCCGAGGAGATCGCT |  |
| 3 | Glyma.12g061600 | Forward | TTTCGGAGGTATTCACGTTTGGCA | 241 |
|  |  | Reverse | ACAAGCAGCAGCTAATTTAGGACTCCA |  |
| 4 | Glyma.08g082900 | Forward | CCTTGGGCTGCGTCTTCCCG | 466 |
|  |  | Reverse | CCGGGGACGAAGTTGGTGGC |  |
| 5 | Glyma13g07610 | Forward | CCATGGCTGGCTTCCCCACG | 443 |
|  |  | Reverse | GCCTGGGGGCTTGTAGGCGA |  |
| 6 | Glyma.07g014500 | Forward | ACAAGGGCAATGCACCGGCA | 311 |
|  |  | Reverse | GCTTTCGCCTCTGTGGCCTCC |  |
